# Co-delivery of Salinomycin and Curcumin for Cancer Stem Cell Treatment by Inhibition of Cell Proliferation, Cell Cycle Arrest, and Epithelial–Mesenchymal Transition

**DOI:** 10.3389/fchem.2020.601649

**Published:** 2021-01-15

**Authors:** Yongmei Zhao, Kaikai Wang, Yuanlin Zheng, Xiaobao Zeng, Yi Chieh Lim, Tianqing Liu

**Affiliations:** ^1^School of Pharmacy, Nantong University, Nantong, China; ^2^Danish Cancer Society Research Center, Copenhagen, Denmark; ^3^NICM Health Research Institute, Western Sydney University, Sydney, NSW, Australia

**Keywords:** nanomedicine, polymeric nanoparticles, curcumin, salinomycin, cancer stem cells

## Abstract

Malignant cancer is a devastating disease often associated with a poor clinical prognosis. For decades, modern drug discoveries have attempted to identify potential modulators that can impede tumor growth. Cancer stem cells however are more resistant to therapeutic intervention, which often leads to treatment failure and subsequent disease recurrence. Here in this study, we have developed a specific multi-target drug delivery nanoparticle system against breast cancer stem cells (BCSCs). Therapeutic agents curcumin and salinomycin have complementary functions of limiting therapeutic resistance and eliciting cellular death, respectively. By conjugation of CD44 cell-surface glycoprotein with poly(lactic-co-glycolic acid) (PLGA) nanoparticles that are loaded with curcumin and salinomycin, we investigated the cellular uptake of BCSCs, drug release, and therapeutic efficacy against BCSCs. We determined CD44-targeting co-delivery nanoparticles are highly efficacious against BCSCs by inducing G_1_ cell cycle arrest and limiting epithelial–mesenchymal transition. This curcumin and salinomycin co-delivery system can be an efficient treatment approach to target malignant cancer without the repercussion of disease recurrence.

## Introduction

Resistance to chemotherapy is the major cause of cancer relapse and mortality. Early indications of intrinsic resistance emphasized on the presence of cancer stem cells (CSCs), which are highly mesenchymal in nature and have the ability to self-renew, differentiate, and promote tumorigenesis. They play an important role in chemotherapy and radiotherapy resistance via enhanced anti-apoptotic, DNA repair, and reactive oxygen species (ROS)-protective mechanisms, often leading to tumor recurrence including metastasis. There is now overwhelming clinical evidence to support enriched CSCs as the dominant population in reviving tumor growth despite initial aggressive chemotherapeutic intervention. Therefore, effective elimination of CSCs is essential to impede tumor relapse and drug resistance to improve patients' survival. However, current treatments may harm the normal stem cell pool and result in severe side effects. Therefore, therapeutics to specifically remove CSCs remain a major challenge for anti-cancer treatment.

Salinomycin is a polyether ionophore antibiotic with efficacy as an anti-cancer drug (Naujokat and Steinhart, 2012). Pre-clinical data indicates salinomycin has the potential to reduce tumorigenicity by targeting CSCs and has been effective in several cancer types (Gupta et al., 2009; Lu et al., 2011; Ketola et al., 2012; Lim et al., 2020). The anti-cancer mechanism of salinomycin is associated with dysregulation of metal ions (Paulus et al., 1998), activation of autophagy-mediated cell death, and inhibition of stem cell maintenance (Lu et al., 2011; Yue et al., 2013; Mai et al., 2017). A recent study found that salinomycin and its derivative, ironomycin, exhibited a potent and selective activity against breast cancer stem cells (BCSCs) by accumulating and sequestering iron to induce ferroptosis, which is a recently described form of iron-dependent cell death marked by the oxidative modification of phospholipid membranes (Mai et al., 2017). However, it is challenging to efficiently deliver salinomycin (Sal) to tumor sites due to its hydrophobicity, unfavorable pharmacokinetic profile, and cytotoxicity during systemic drug administration (Naujokat and Steinhart, 2012). Curcumin (Cur) is a bioactive ingredient derived from the root of *Curcuma longa L*, which have been widely used in the prevention and treatment of a number of diseases, including cancer (Sharma et al., 2005; Zhou et al., 2011; Shanmugam et al., 2015; Gao et al., 2017; Motevalli et al., 2019). Curcumin is a potent tumor suppressor as it can inhibit tumor growth at cellular and *in vivo* level. Although the mechanism is not fully understood, studies have shown that curcumin can lead to inhibition of cancer cell invasion (Baek et al., 2003; Shao et al., 2017), cell cycle arrest, and induction of autophagic cell death. More recently, researchers have investigated the anti-metastasis potentials of curcumin (Basnet and Skalko-Basnet, 2011; Norris et al., 2013; Teiten et al., 2014; Kotcherlakota et al., 2016) and found it can inhibit CSCs' viability and their stemness via ROS and the signal transducer and activator of transcription 3 (STAT3) signaling pathway (Buhrmann et al., 2020). However, its therapeutic potential is limited due to its rapid metabolism and poor solubility and absorption (Tsai et al., 2011).

Nanomedicines provide new strategies in cancer targeting and treatment due to high drug loading capability, cancer-targeting ability, and even the potential to overcome the efflux pump-mediated drug resistance by delivering a high concentration of intracellular drugs (Cho et al., 2008). Our previous studies showed that conjugation of salinomycin with gold nanoparticles can efficiently induce ferroptotic cell death of BCSCs by increasing the generation of ROS, mitochondrial dysfunction, and lipid oxidation with higher iron accumulation and GPX-4 inactivation (Zhao et al., 2019). Here, the combination of salinomycin and curcumin was used to achieve synergetic effect in treating BCSCs. Salinomycin and curcumin were loaded onto poly(lactic-co-glycolic acid) (PLGA) polymeric nanoparticles via double emulsion method to form nanoparticles (Avgoustakis et al., 2002; Alam et al., 2017). Additionally, hyaluronic acid (HA) was also conjugated to nanoparticles as targeting moiety due to its specific interaction with the CD44 receptor, which is a cell surface glycoprotein and commonly overexpressed on BCSCs (Zöller, 2011; Ganesh et al., 2013; Sanfilippo et al., 2020). The CD44-targeting nanoparticles loaded with salinomycin and curcumin showed improved therapeutic efficiency against BCSCs by inducing G_1_ cell cycle arrest and limiting epithelial–mesenchymal transition (EMT).

## Materials and Methods

### Materials

Dulbecco's Modified Eagle Medium (DMEM), fetal bovine serum (FBS), penicillin–streptomycin, propidium iodide, Triton X-100, PageRuler Prestained Protein Ladder, and nitrocellulose membrane were purchased from Thermo Fisher. Curcumin, salinomycin, 1-ethyl-3-(3-dimethylaminopropyl)carbodiimide hydrochloride (EDC), and N-hydroxysuccinimide (NHS) were bought from Sigma-Aldrich and used directly without any purification. Mouse anti-vimentin, mouse anti-E-cadherin, and rabbit anti-β-actin primary antibodies purchased from Abcam. Odyssey blocking buffer, IRDye® 800CW goat anti-mouse secondary antibody, and IRDye® 680RD goat anti-rabbit secondary antibody were purchased from LI-COR.

### Nanoparticle Synthesis and Surface Functionalization

NH2-PEG-NH2 (MW: 3.4 k), PLGA (MW: 10 k), and PLGA-PEG-NH2 (MW: 18.6 k) co-polymer were bought from Meiluo Tech Company. Curcumin and salinomycin were loaded into the PLGA-PEG-NH2 copolymer via the well-established double emulsion method (Avgoustakis et al., 2002). Briefly, curcumin (1 mg/ml) and salinomycin (molar ratio of Cur and Sal = 1:1) were dissolved in organic phase containing co-polymer (1.75%, *w*/*v*), and the mixture was mixed using an ultrasonic homogenizer (LABSONIC M, Sartorius, Germany). The primary particles were added into a PVA solution (10 ml, 2%, *w*/*v*), and the mixture was sonicated again. The free drugs were then removed by centrifugation at 20,000 rpm for 15 min and washed with water. The formed nanoparticles were labeled as PEG-PLGA-Cur-Sal.

After drug loading, targeting moiety HA was conjugated onto the polymeric nanoparticles via EDC coupling. Briefly, desalted HA (1 equivalent), EDC (3 equivalents), and NHS (3 equivalents) were dissolved in 2-(N-morpholino)ethanesulfonic acid (MES) buffer and stir in ice bath to activate carboxylic group for half an hour. PEG-PLGA-Cur-Sal was then added and the mixture was stirred for another 4 h at room temperature to form nanoparticles named HA-PEG-PLGA-Cur-Sal. They were purified by using centrifugation at 20,000 rpm for 15 min.

### Characterization of the Nanoparticles

^1^H NMR spectra were acquired on a Bruker AVANCE 400 MHz spectrometer. A gel permeation chromatography—multi-angle laser light scattering (GPC-MALLS) system consisting of a 1,515 isocratic pump (Waters), Styragel HT 6E and Styragel HT 3 columns (Waters), 2,414 differential refractive index detector (Waters), and a DAWN HELEOS laser light scattering detector (Wyatt) with THF as an eluent at a flow rate of 1 ml/min was used to measure the molecular weight and dispersity of the polymers. After drug loading, the size of the nanoparticles was measured via dynamic light scattering (DLS) to measure the hydrodynamic diameter using a Malvern Zetasizer, and a Philips CM100 transmission electron microscope (TEM) was also used to observe their morphology.

### Cancer Stem Cell Culture

MCF-7 cells were bought from ATCC (USA) and cultured in DMEM medium containing 10% (*v*/*v*) FBS and 1% (v/*v*) penicillin–streptomycin. They were kept in a humidified 37°C incubator with 5% CO_2_. After serum-free treatment, BCSC sub-populations in the MCF-7 cells were isolated using MagCellect Human BCSC isolation kit (R&D Systems) according to protocols from the manufacturer. In brief, MCF-7 cells (1 × 10^7^ cells/ml) were prepared in buffer and mixed with anti-human CD24 biotinylated antibody and then Streptavidin Ferrofluid for selection of CD24^low^ cells. Those cells were subsequently incubated with a biotinylated anti-human CD44 antibody and Streptavidin Ferrofluid for selection of CD44^high^ cells. After washing, magnetically tagged CD24^low^/CD44^high^ BCSCs were isolated through the magnet. The cells were directly treated with drug-loaded nanoparticles and control samples after isolation.

### Drug Loading Efficiency and Drug Release Study

Salinomycin loading efficiency was analyzed by using a UV-Vis-NIR spectrophotometer following a published method (Wang et al., 2014). The drug concentration of curcumin was calculated using the linear portion of the calibration curve obtained by the UV spectrophotometer at 427 nm for serial drug concentrations.

### Cellular Uptake Assay With Flow Cytometry

Flow cytometry was used to quantify cellular interaction following incubation of the cells with each treatment group. BCSCs were seeded in six-well culture plates (2 × 10^5^ cells/well). Fluorescent dye Cy5.5 was encapsulated in the nanoparticles (NPs) to image cellular uptake. After pre-incubation for 24 h, cells were treated with HA-PEG-PLGA NPs and PEG-PLGA NPs, respectively. After 6 h, the cells were washed with PBS twice and cells of each well were collected and imaged using a FACSCanto A flow cytometer (BD Bioscience, USA). Fluorescent dye coumarin-6 was encapsulated in the NPs, and cellular uptake was monitored using a Leica TCS SP8 CARS confocal microscope (Leica, Germany) after 4-h incubation of untargeted PEG-PLGA NPs and targeted HA-PEG-PLGA NPs.

### Proliferation Assay

Cells were seeded at 800 cells per well in 96-well plates and cultured until they reached ~10% confluence. Cells were then treated overnight with various concentrations of drug-loaded nanoparticles or controls. Cell proliferation was monitored using the Incucyte Zoom (Essen, MI, USA). Images were taken at regular time intervals over 6 days, and cell growth curves were plotted with the Incucyte Zoom software.

### Cell Cycle G1/S Assessment Using Flow Cytometry

Cells were seeded onto six-well plates and cultured overnight until they reached 80% confluence. After a subsequent 24-h treatment with drug-loaded nanoparticles or the relevant control, the cells were treated with 300 μl of 1 mg/ml propidium iodide, 0.1% (*v*/*v*) Triton X-100, and 0.8% (*v*/*v*) RNAse A solution for 30 min at room temperature. Cells were washed with PBS and harvested for cell cycle measurements while protected from light. Single cells were identified by measurement of forward and side scatter. Then, doublets were removed using pulse processing. Measurements took place using a B670LP-A filter on a FACSCanto A flow cytometer.

### Wound Scratch Assays

Cell migration was assessed using an established wound scratch assay (Fan et al., 2011). Cells were seeded at 64,000 cells per well in 96-well plates and incubated until they reached more than 80% confluence. The plates were placed in an Incucyte wound maker (Essen, MI, USA) to ensure consistency in the wounds made on the cell monolayers in each well. The medium and cell debris were then removed and replaced with 200 μl of fresh medium containing drug-loaded nanoparticles or controls. The plates were placed in the Incucyte Zoom (Essen, MI, USA), and the wound width was monitored over 24 h for later analysis using Incucyte Zoom software.

### Cell Attachment

Cells were seeded onto six-well plates and left to attach overnight until over 80% confluence and then treated with drug-loaded nanoparticles or controls for 24 h. The cells were washed with PBS and then detached using versene. They were then seeded 5 × 10^4^ cells per well onto gelatin-precoated 96-well plates for 2 h. After washing with PBS and fixation using 4% PFA for 15 min, the cells were imaged using the Incucyte Zoom (Essen, MI, USA) and the number of cells attached was quantified using Incucyte Zoom software.

### Western Blotting

The expression of EMT markers was assessed by western blotting. Cells were seeded onto six-well plates and cultured overnight until they reached over 80% confluence. They were then treated with drug-loaded nanoparticles or controls for 24 h, washed with PBS, and harvested with versene. Ice-cold lysis buffer was added to each cell pellet for 15 min, and protein supernatants were obtained after spinning at 16,000G for 15 min at 4°C. The total protein concentration in each extract was measured using the bicinchoninic acid protein assay.

Protein samples were diluted using Milli-Q water to give a concentration of 50 μg protein/20 μl. Loading buffer (5 μl) was added to each sample, and samples were heated at 75°C for 10 min. Samples were loaded into wells of 15% acrylamide gels with a protein ladder and run at 100 V. The proteins were subsequently transferred onto nitrocellulose membranes at 100 V for 1 h, then blocked with 5 ml of blocking buffer at 4°C overnight. Blots were treated with a mouse anti-vimentin monoclonal antibody (1:1,000) or a mouse anti-E-cadherin monoclonal antibody (1:1,000) in blocking buffer for 1 h at room temperature on a shaker, while a rabbit anti-β-actin monoclonal antibody was used as a loading control (1:1,000). After washing with 1X TBST four times every 15 min for 1 h, the blots were incubated with goat anti-mouse polyclonal secondary antibodies (1:5,000) in the dark for 1 h. After washing for four times and drying, the membranes were scanned with the Odyssey CLx (LI-COR, NE, USA) imaging system and data were analyzed with Image Studio software.

### Statistical Analysis

Multiple group comparisons were carried out using *T*-test, using GraphPad Prism version 7. This enabled significance against DMSO controls to be calculated. All results are shown as mean ± SEM of at least three replicates.

## Result and Discussion

### Nanoparticle Preparation and Drug Loading

PLGA-PEG-NH_2_ co-polymer was validated via ^1^H NMR spectrum (Supplementary Figure 2). PLGA-PEG-NH_2_ co-polymer showed both the characteristic peak of PLGA (methyl group: 1.44–1.83 ppm and multiple peaks: 5.17–5.40 and 4.66–4.89 ppm) and the typical peak of PEG in which the methane proton is around 3.62 ppm (CH_2_-CH_2_-O) and validated the successful conjugation of PLGA-PEG-NH_2_. PLGA-PEG nanoparticles loaded with salinomycin and curcumin were prepared using the well-established double emulsion solvent evaporation method (Avgoustakis et al., 2002; You et al., 2018). HA was conjugated onto nanoparticles via EDC coupling and confirmed by hexadecyltrimethylammonium bromide (CTAB) turbidimetric method (Chen and Wang, 2009; Song et al., 2009). The hydrodynamic size of the nanoparticles increased from 120.1 ± 5.5 to 153.4 ± 4.6 nm after conjugation with HA, which was measured by dynamic light scattering (Table 1). Nanoparticle morphology was confirmed by TEM (Supplementary Figure 1). The surface charge also exhibited a negative zeta potential value after HA conjugation because of the negative carboxylic moiety of HA, which confirmed the conjugation of HA onto the particles. Approximately 70% of salinomycin and 82% of curcumin were loaded in polymeric nanoparticles, characterized by measuring UV-Vis spectra. Free salinomycin and curcumin molecules were separated from the solution and subsequently removed by centrifugation.

**Table 1:** Hydrodynamic size and drug loading of the nanoparticles.

**Sample**	***D*_**h**_ (nm)^a^**	**Zeta potential (mV)**	**Sal loading (%)^b^**	**Cur loading (%)^b^**
PEG-PLGA-Cur-Sal	120.1 ± 5.5	−3.1 ± 0.2	70	82
HA-PEG-PLGA-Cur-Sal	153.4 ± 4.6	−32.6 ± 2.5	–	–

a *Determined by DLS*.

b *Determined by UV-Vis spectroscopy*.

### Drug Release Study

**Figure 1:**
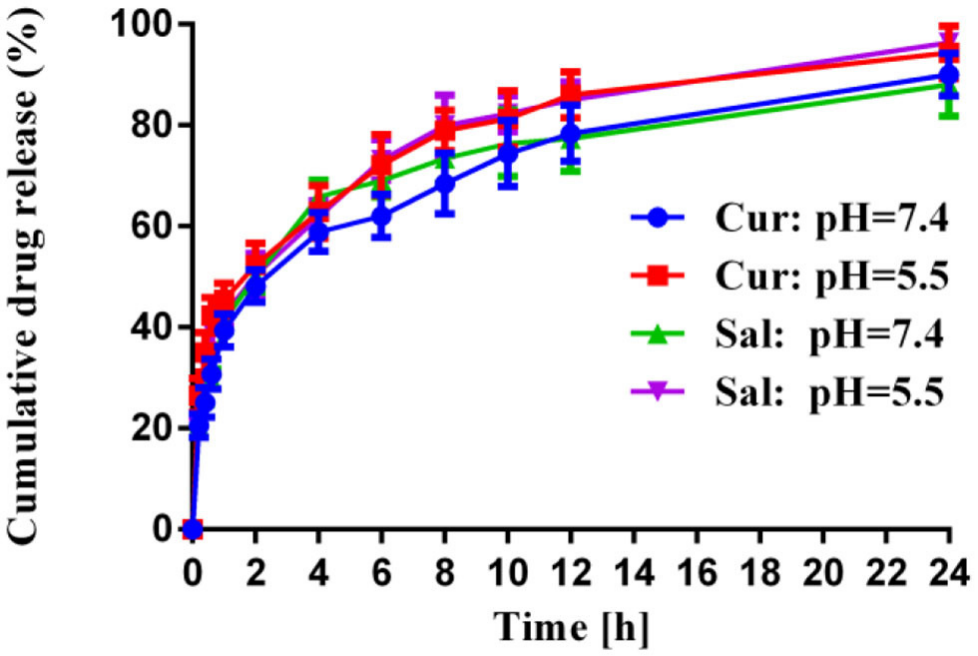
*In vitro* release profiles of Cur and Sal from HA-PEG-PLGA-Cur-Sal nanoparticles in sodium phosphate buffer at pH 5.0 and pH 7.4 at 37°C, respectively. Error bars show mean ± standard deviation from experiments performed in triplicate.

The kinetics of the drug release was evaluated over time upon their *in vitro* incubation in PBS buffer at 37°C, and the results are summarized in Figure 1. The higher hydrophilic moiety of PEG amount in co-polymer contributed to greater drug release. During the initial hours, there was a rapid release that led to a higher loading of salinomycin and curcumin in the vicinity of the surface and faster drug diffusion (Panagi et al., 2001; Wang et al., 2013). After 12 h, levels of salinomycin and curcumin were observed as a sustained curve, possibly due to the slow release of drugs within the polymeric matrix. Post-24 h, ~88% of salinomycin and 90% of curcumin were released from polymeric nanoparticles when pH was 7.4. When pH was reduced to 5.0, ~96% of salinomycin and 94% of curcumin were released. This data suggests both salinomycin and curcumin can be released from the polymeric nanoparticles under a similar condition and is expected when coming in contact with tumor tissues.

### *In vitro* CD44-Mediated Cellular Uptake

**Figure 2:**
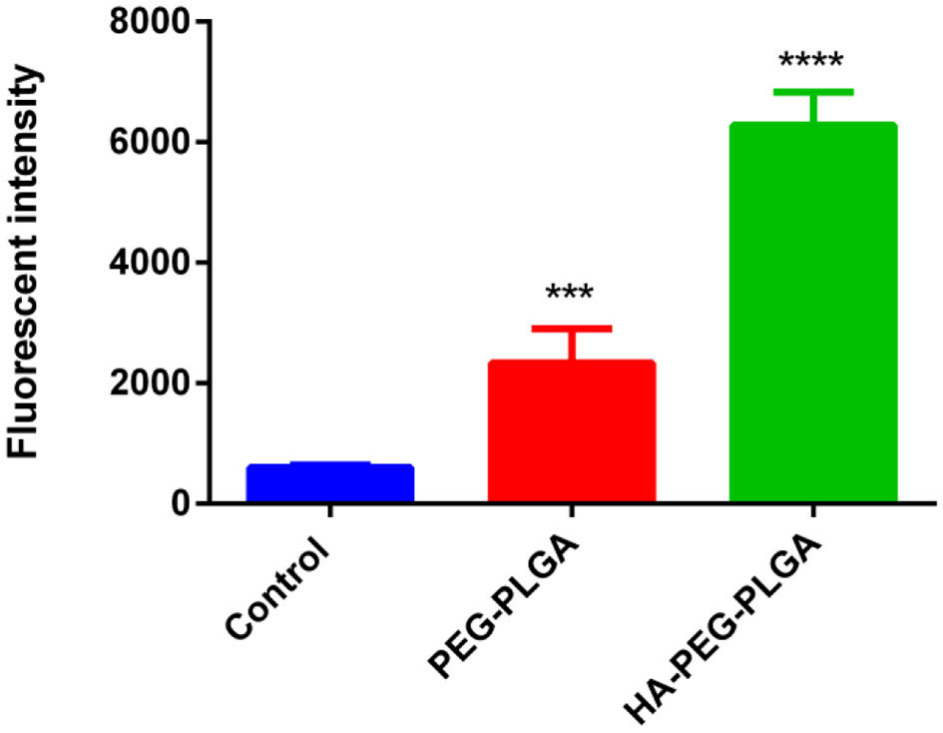
Cell uptake of HA-PEG-PLGA NPs and PEG-PLGA NPs by flow cytometry. The BCSCs were incubated with different formulations for 6 h. Data are mean ± SD (*n* = 3) (***p < 0.001, ****p < 0.0001).

Flow cytometry analysis was used to determine the internalization of HA-PEG-PLGA NPs by cancer cells and if this correlation is associated with the over-expression of CD44 receptor. BCSCs treated with HA-PEG-PLGA NPs showed a higher fluorescence intensity than BCSCs incubated with PEG-PLGA NPs after 6 h. This is due to a HA receptor that leads to cellular internalization via HA receptor-mediated endocytosis. As shown in Figure 2, the cellular association of HA-PEG-PLGA NPs was approximately three times greater than non-specific PEG-PLGA NPs at 37°C. This suggests HA-PEG-PLGA NPs could enter into the cell membrane and efficiently target cells by binding with CD44 that is overexpressed in BCSCs. HA targeting ability was also confirmed by confocal fluorescence micrograph of MCF-7 cells after 4-h incubation of untargeted PEG-PLGA NPs and targeted HA-PEG-PLGA NPs at the same concentration of coumarin-6. Significant fluorescence signal of coumarin-6 was observed in HA-PEG-PLGA NPs (Supplementary Figure 3).

### *In vitro* Cell Proliferation and Viability

**Figure 3:**
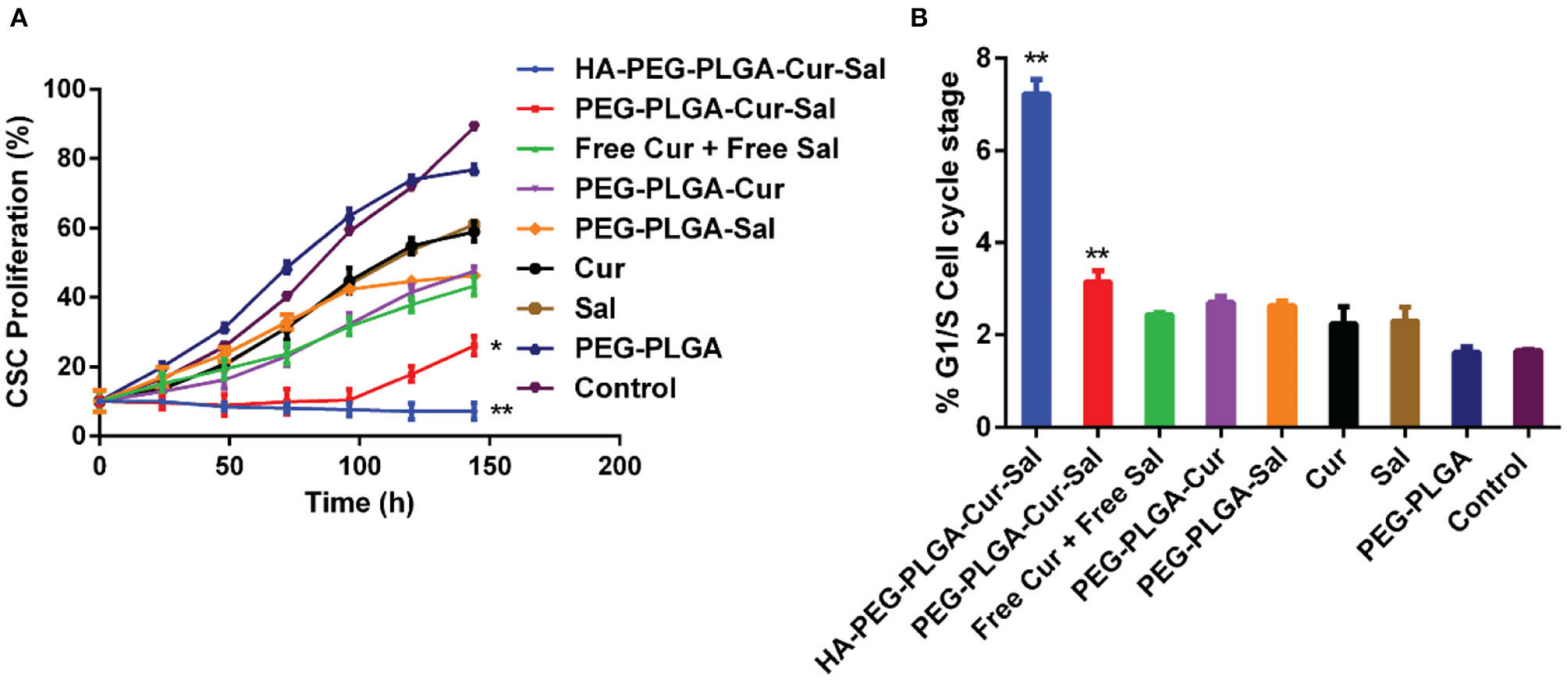
**(A)** Cell proliferation study of HA-PEG-PLGA-Cur-Sal, PEG-PLGA-Cur-Sal, PEG-PLGA-Cur, PEG-PLGA-Sal, PEG-PLGA, Cur, and Sal against CSCs. **(B)** Cell cycle arrest study of HA-PEG-PLGA-Cur-Sal, PEG-PLGA-Cur-Sal, PEG-PLGA-Cur, PEG-PLGA-Sal, PEG-PLGA, Cur, and Sal against CSCs. Values are means ± standard deviations (*n* = 5, S.D.) (*p < 0.05, **p < 0.01).

The *in vitro* cytotoxicity analysis was grouped as follows: HA-PEG-PLGA-Cur-Sal, PEG-PLGA-Cur-Sal, PEG-PLGA-Cur, PEG-PLGA-Sal, PEG-PLGA, Cur, and Sal. These compounds were evaluated against BCSCs by using cell proliferation assay with an incubation period of 144 h. The cytotoxicity data of BCSCs summarized in Figure 3A show the different formulations using a series of eight different concentrations of Sal and each group received the same corresponding Sal concentration. Cur was kept at a constant concentration. From Figure 3A, HA-PEG-PLGA-Cur-Sal, which has a specific affinity with over-expressed CD44 receptor, showed the greatest cytotoxicity as compared to the remaining formulations at the same drug concentration. Additionally, PEG-PLGA-Cur-Sal showed increased cytotoxicity compared to formulations containing either Cur or Sal. ED50 values were derived as described by Tallarida (2011), and synergism was observed in both HA-PEG-PLGA-Cur-Sal and PEG-PLGA-Cur-Sal that exhibited a super-additive effect.

### Cell Cycle Arrest Study

Chemotherapeutic drugs are highly toxic to the progression of cell cycle and thus can promote cellular death. To determine whether the drugs and their formulations cause cell cycle arrest, we investigate cell cycle progression by flow cytometry. The G1 phase of cell cycle is the initial cell cycle phase in which cells progress for subsequent duplication of itself. As shown in Figure 3B, both HA-PEG-PLGA-Cur-Sal and PEG-PLGA-Cur-Sal can significantly prolong the G_1_/S phase of the cell cycle, while HA-PEG-PLGA-Cur-Sal promoted G_1_/S arrest to a greater extent than the PEG-PLGA-Cur-Sal group. This suggests that HA-PEG-PLGA-Cur-Sal is the most effective in preventing BCSCs from subsequent progression into S-phase in which the genome duplication occurs. This indicates that co-delivery of salinomycin and curcumin causes extensive G_1_/S arrest, leading to the subsequent activation of apoptosis.

### Cell Migration and EMT

**Figure 4:**
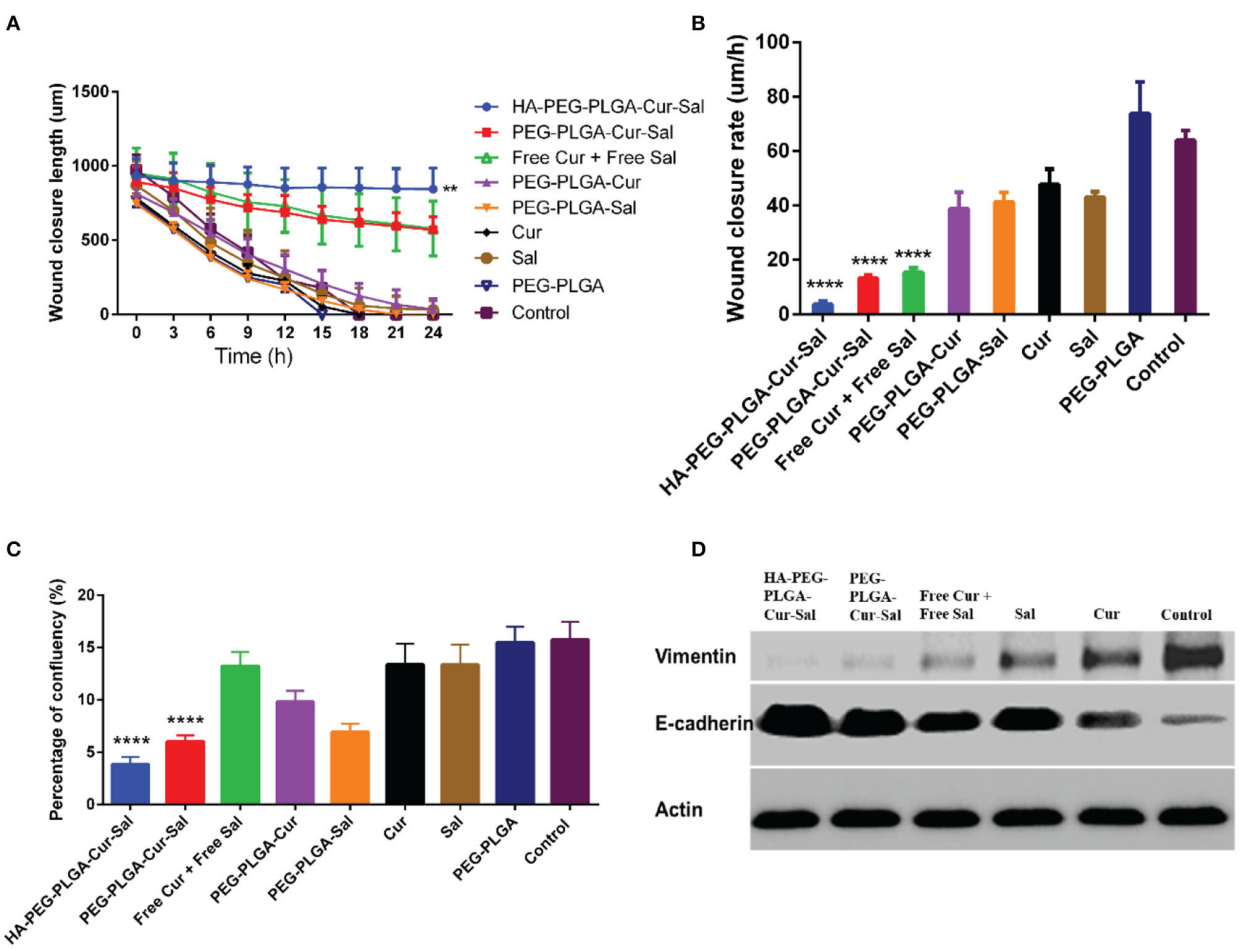
Cell migration behavior after treatment of PEG-PLGA-Cur, PEG-PLGA-Sal, PEG-PLGA, Cur, and Sal against CSCs using wound healing assay: **(A)** wound closure length, **(B)** wound closure rate, and **(C)** percentage of confluency of wound healing assay. **(D)** Expression of epithelial–mesenchymal transition markers of HA-PEG-PLGA-Cur-Sal, PEG-PLGA-Cur-Sal, free Cur + free Sal, Cur, and Sal. Values are means ± standard deviations (*n* = 5, S.D.) (**p < 0.01, ****p < 0.0001).

BCSCs play a pivotal role in migration, which is essential for the pathogenesis of cancer during invasion and metastasis. Conversely, lack of cellular migration can lead to a failure in regenerative therapies. To validate cell migration *in vitro*, we adopted wound-healing assay which is widely used as a model to determine cell migration *in vivo*. As shown in Figures 4A,B, BCSCs treated with HA-PEG-PLGA-Cur-Sal showed significant reduction of cell migration in both wound length and rate of wound closure when compared to other treated groups. The lack of migration has a direct impact on cancer cell invasion and tumor metastasis. In Figure 4C, data indicated BCSCs that received HA-PEG-PLGA-Cur-Sal treatment had the lowest cellular confluency during cell attachment assay due to its reduced adhesion ability of BCSCs. Figure 4D shows that the immunoblotting of vimentin (mesenchymal marker) and E-cadherin (epithelial maker) confirmed HA-PEG-PLGA-Cur-Sal could reverse the properties of EMT, thereby limiting BCSCs to migrate.

## Conclusion

In summary, PLGA nanoparticle delivery system co-loaded with anticancer drug Sal and Cur was successfully synthesized and functionalized with HA as a targeting moiety. BCSCs showed higher sensitivity to this formulation with a combination of Sal and Cur by not only efficiently inducing cell death but also inhibiting cell migration and attachment. Our study revealed HA-PEG-PLGA-Cur-Sal could impede G1 phase cell cycle progression, leading to cell cycle arrest and subsequent induction of induced apoptosis of BCSCs. Collectively, our findings suggest that the combination HA-PEG-PLGA-Cur-Sal is an efficient therapeutic strategy against BCSCs and has the potential of treating tumor metastasis and improve patient outcome.

## Data Availability Statement

The raw data supporting the conclusions of this article will be made available by the authors, without undue reservation.

## Author Contributions

YZha: methodology and data interpretation and writing—original draft preparation. KW: methodology and data interpretation. YZhe: experiments and data collection. YL: methodology validation and review. TL: conceptualization, supervision, and manuscript editing. XZ: revision of manuscript. All authors contributed to the article and approved the submitted version.

## Conflict of Interest

The authors declare that the research was conducted in the absence of any commercial or financial relationships that could be construed as a potential conflict of interest.
