# Supplementary material for: Co-delivery of Salinomycin and Curcumin for Cancer Stem Cell Treatment by Inhibition of Cell Proliferation, Cell Cycle Arrest, and Epithelial–Mesenchymal Transition
Source: Front Chem. 2021 Jan 15;8:601649. doi: 10.3389/fchem.2020.601649 (PMC7843432; doi:10.3389/fchem.2020.601649)
Supplement: Supplementary file 1 [file Data_Sheet_1.pdf]

Co-delivery of salinomycin and curcumin for cancer stem cell treatment by inhibition of cell proliferation, cell cycle arrest and epithelial–mesenchymal transition

Yongmei Zhao[1#], Kaikai Wang[1#], Yuanlin Zheng[1], Xiaobao Zeng[1], Yi Chieh Lim[2], Tianqing Liu[3*]

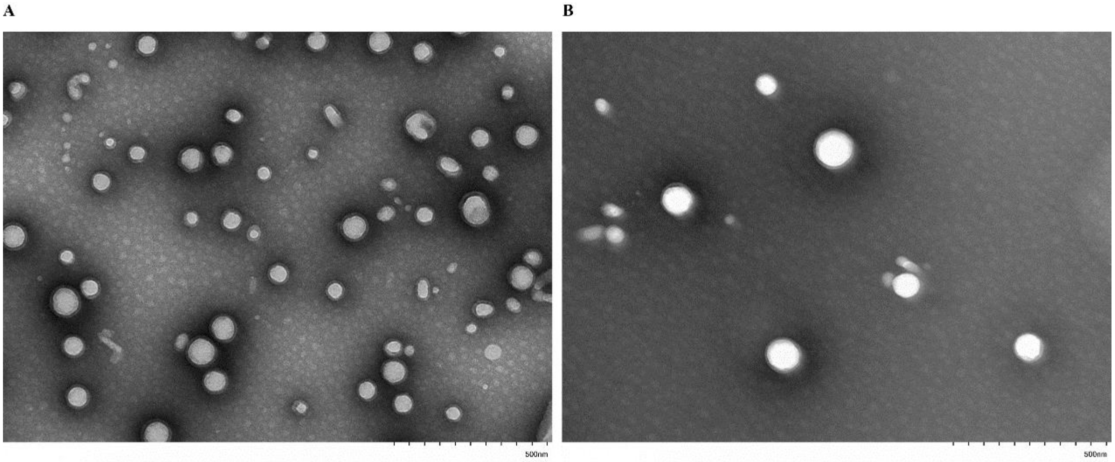

Figure S1. Characterization of nanoparticle: (A) TEM imaging of PEG-PLGA-Cur-Sal, and (B) TEM imaging of HA-PEG-PLGA-Cur-Sal.

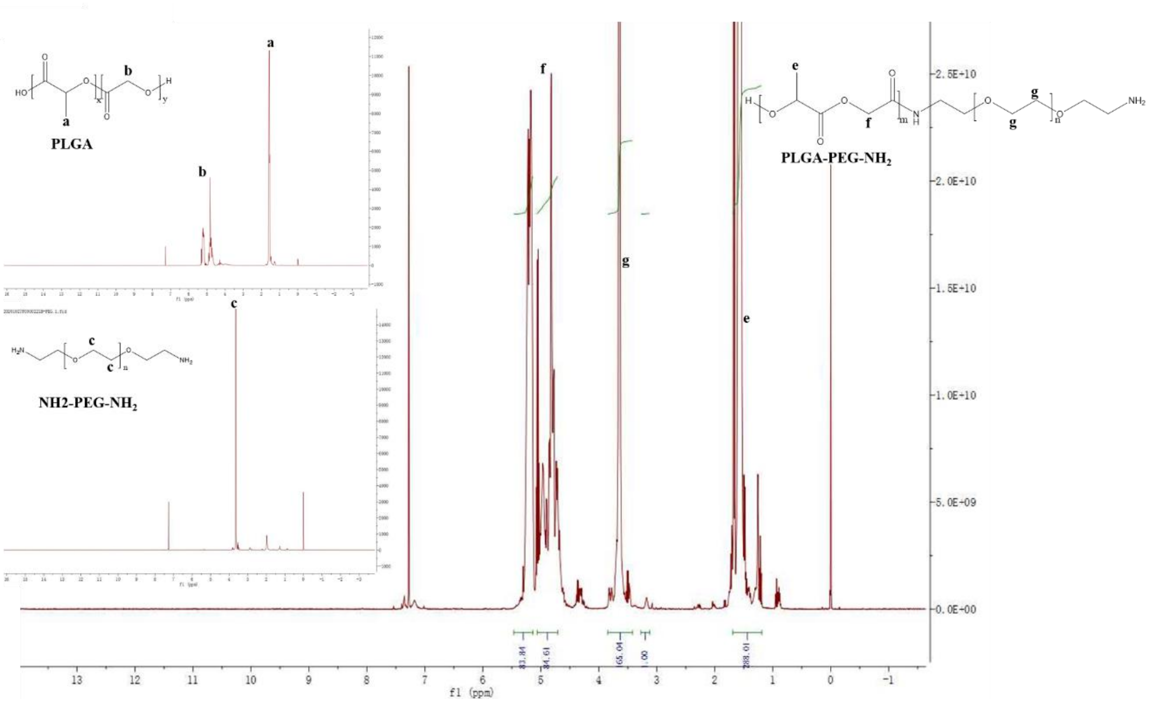

Figure S2. 1H NMR spectrum of PLGA-PEG-NH2.

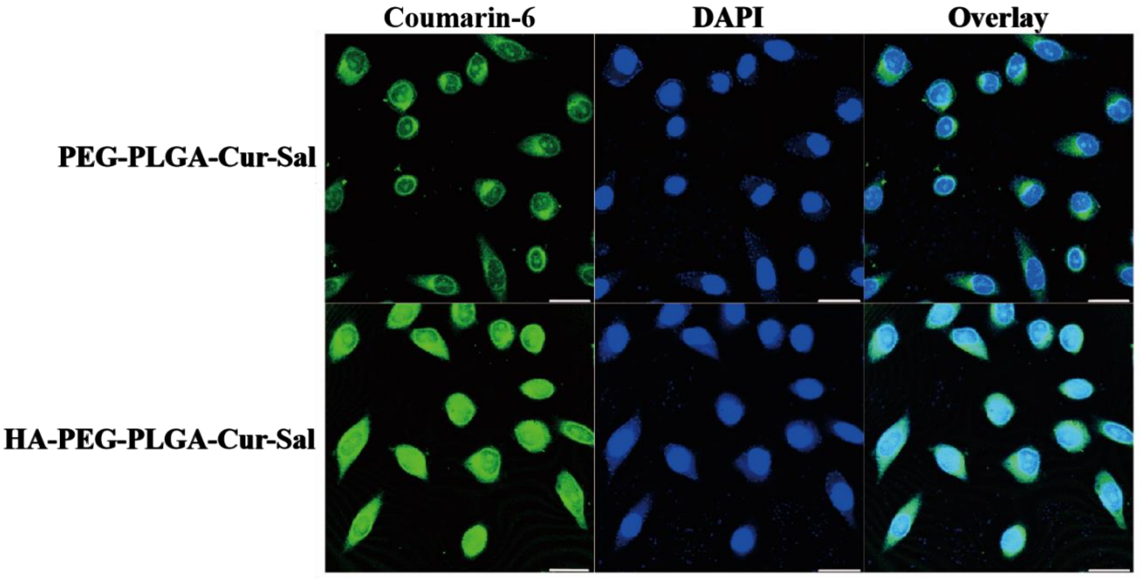

Figure S3. Confocal fluorescence micrographs of MCF-7 cells after 4 h incubation in same concentration of coumarin-6: (A) Untargeted PLGA-SAL-CUR, and (B) Targeted HA-PLGA-SAL-CUR. Scalebar denotes 25 μm.
